# The Transcriptional Landscape of *Campylobacter jejuni* under Iron Replete and Iron Limited Growth Conditions 

**DOI:** 10.1371/journal.pone.0079475

**Published:** 2013-11-01

**Authors:** James Butcher, Alain Stintzi

**Affiliations:** Ottawa Institute of Systems Biology, Department of Biochemistry, Microbiology and Immunology, University of Ottawa, Ottawa, Ontario, Canada; Iowa State University, United States of America

## Abstract

The genome-wide *Campylobacter jejuni* transcriptional response under iron replete and iron limited conditions was characterized using RNA-seq. We have identified 111 novel *C. jejuni* 5’UTRs and mapped 377 co-transcribed genes into 230 transcriptional operons. In contrast to previous microarray results, the *C. jejuni* iron stimulon is less extensive than previously believed and consists of 77 iron activated genes and 50 iron repressed genes. As anticipated, the iron repressed genes are primarily those involved in iron acquisition or oxidative stress defense. Interestingly, these experiments have revealed that iron is an important modulator of flagellar biogenesis with almost all the components of the flagella found to be iron activated. Given that motility is a well-known *C. jejuni* colonization factor, this suggests that there is an important regulatory coupling of flagellar biogenesis and iron level in *C. jejuni*. In addition we have identified several consensus mutations in the *C. jejuni* NCTC11168 strain that are widespread in the *Campylobacter* research community and which may explain conflicting phenotypic reports for this strain. Comparative analysis of iron responsive genes with the known Fur regulon indicates that many iron responsive genes are not Fur responsive; suggesting that additional iron regulatory factors remain to be characterized in *C. jejuni*. Further analysis of the RNA-seq data identified multiple novel transcripts including 19 potential ncRNAs. The expression of selected ncRNAs was confirmed and quantified with qRT-PCR. The qRT-PCR results indicate that several of these novel transcripts are either Fur and/or iron responsive. The fact that several of these ncRNAs are iron responsive or Fur regulated suggests that they may perform regulatory roles in iron homeostasis.

## Introduction

Iron is crucial for microbial growth as it forms the active center of a variety of diverse enzymes involved in essential cellular processes such as energy metabolism and redox reactions [1]. While iron is one of the most abundant metals on earth, it is mostly present in the ferric form which is insoluble under neutral pH and aerobic conditions. Microorganisms have thus evolved elaborate strategies to acquire iron from their environment. Pathogens such as *Campylobacter jejuni* face additional challenges in acquiring sufficient iron as their hosts typically restrict free iron to prevent unwanted microbial growth [2]. Pathogens subvert this defense system by expressing multiple iron acquisition pathways under conditions of iron limitation. On the other hand, acquiring excess amounts of iron poses its own metabolic risks. Excess iron levels are toxic to most microorganisms by triggering oxidative stress through Fenton chemistry [1,2]. Thus microorganisms must carefully regulate iron metabolism to ensure that they acquire sufficient iron to grow yet avoid iron toxicity [1,2]. 

The classical iron regulatory protein in most Gram-negative bacteria is the ferric uptake regulator (Fur) protein [1,2]. Fur senses intracellular iron and represses genes involved in iron acquisition when cellular iron levels rise. In addition, many microorganisms also contain small regulatory RNAs that are involved in regulating iron metabolism. The most studied of these sRNAs is *E. coli* RyhB [3]. This small regulatory RNA is repressed by Fur under iron-replete conditions. However under iron limited conditions, Fur repression is relieved and RyhB can be transcribed, and together with the Hfq RNA binding protein, target its downstream effectors [3]. RyhB targets the mRNA transcripts of several non-essential, iron-binding proteins and promotes their degradation. This iron sparing response ensures that iron is preferentially used for essential cellular reactions [3]. The combination of these two systems ensures that iron levels are appropriately controlled and that iron is preferentially used for essential pathways. Indeed, pathogens such as that are missing these regulators, including *C. jejuni*, *H. pylori*, *V. cholerae* and *S. flexneri*, are defective in controlling iron metabolism and show decreased virulence and colonization *in vivo* [4-9]. 

Previous work has characterized the transcriptomic response of *C. jejuni* to iron limitation using genome wide microarray analysis [4,10]. These microarray studies found extensive remodelling of the *C. jejuni* transcriptome under iron limitation and greatly contributed to our current understanding of *C. jejuni* iron homeostasis. However while microarray studies are instructive, they are being increasingly eclipsed by the utility of next generation high throughput sequencing (HTS) technologies [11]. Several studies have indicated that HTS technologies can determine gene expression level more accurately than microarrays [12,13], and also offer exciting insight into the transcript structures present under each condition studied [11,14,15]. The RpoN regulon of *C. jejuni* has been characterized by profiling the transcriptome of an *rpoN* mutant using HTS and compared to results previously obtained using microarray technologies [16]. Bioinformatic analysis of the transcriptomic data revealed numerous potential novel transcripts and detected possible transcriptional activity originating from these genomic regions. More recently, the transcriptomes of several *C. jejuni* strains have been profiled using a differential RNA-sequencing (dRNA-seq) technology that allows for the enrichment of unprocessed RNA species [17]. This approach has greatly improved our understanding of transcriptional start sites (TSS) in *C. jejuni* and also provided experimental evidence for several potential ncRNAs.

We report herein the first HTS characterization of *C. jejuni*’s transcriptomic response to iron limitation. Moreover, we have used both mRNA and sRNA specific HTS libraries to fully capture the fully transcriptomic potential of *C. jejuni*. 

## Methods

### Bacterial Strains and growth

*C. jejuni* NCTC11168 and the Δ*fur* isogenic mutant were routinely cultured on Mueller-Hinton (MH) agar plates under microaerophilic conditions at 37°C in a MACS-VA500 workstation (Don Whitley, West Yorkshire, England). To note, our *C. jejuni* NCTC11168 strain is motile and capable of colonizing chicks in contrast to the genome sequenced strain [4,18]. Colonies were picked and grown overnight in MH agar overlaid with MH broth (biphasic cultures). Overnight cultures were washed in minimal essential media (MEMα) (Invitrogen) and used to inoculate 50 mL MEMα supplemented with 10 mM pyruvate. Freshly prepared FeSO_4_ was added at a final concentration of 40 µM as needed to generate iron replete growth conditions. 

### Total RNA Isolation

*C. jejuni* was grown either in iron replete (+40 µM FeSO_4_) or iron limited (-40 µM FeSO_4_) MEMα. *C. jejuni* was innoculated at an intital OD_600_ of 0.05 and grown until midlog phase was reached (OD_600_ 0.2) which normally took ~5-6 hours. Total RNA was then isolated as described previously [4]. Briefly, 10% cold RNA stop solution was added to the cultures (10% buffer saturated phenol in absolute ethanol) and cells were pelleted by centrifugation at 6000 x g. Cells were resuspended in TE buffer and RNA extracted using a hot-phenol chloroform method. RNA was precipitated overnight and the RNA pellet was washed five times in 80% ethanol. RNA pellets were resuspended in TE buffer and treated with DNase I (Epicentre) to remove contaminating genomic DNA. Treated samples were purified using the RNeasy kit (Qiagen) and PCR was performed to confirm the absence of genomic DNA. RNA purity and quality was assessed using a Nanodrop and the Experion RNA StdSens analysis kit (Bio-Rad). Samples with high quality 260/280 and 260/230 ratios and an 18S/16S ratio greater than 1.4 were selected for Illumina sequencing library construction. 

### Illumina library construction and sequencing

Library construction, cluster generation and sequencing were performed at the Beijing Genomics Institute. In addition, ribosomal RNAs were depleted using the RiboMinus^TM^ transcriptome isolation kit from Life Technologies. Each library consisted of a 1:1 mixture of RNA extracted from two biological replicates grown and isolated separately. Library construction and cluster generation was done using standard Illumina protocols with sequencing performed on an Illumina HiSeq 2000 sequencer. The mRNA libraries were subjected to paired-end sequencing with a target read length of 100 nucleotides. Approximately ~6.5 million high quality reads were obtained per library (Table S1), with two sequencing reactions for iron replete conditions and three for iron limited conditions. The miRNA libraries were constructed using the standard protocol for Illumina miRNA sequencing libraries. This included size fractioning the Total RNA and selecting for fragments of ~20-30 nt. The miRNA libraries were subjected to single end sequencing with a target read lengh of 75 nucleotides and resulted in ~20 million reads per library (Table S1) with one sequencing reaction each for iron replete/limited conditions. The Illumina reads were submitted to the Sequence Read Archive at NCBI with the accession numbers SRX327750 and SRX327869 for the mRNA and miRNA experiments respectively.

### Read alignment to the *C. jejuni* genome

Paired end reads were aligned to the *C. jejuni* NCTC11168 genome (NC002163) using Bowtie v0.12.7 [19] with the best flag enabled and with 4 bp trimming on each read’s 3’ end to remove error prone bases. Approximately 90% of reads aligned for each sequencing run. Aligned reads were visualized using Artemis with the Bamview plugin [20]. Reads corresponding to the miRNA libraries were first trimmed to remove barcode and primer sequences and then aligned with Bowtie in single read alignment mode. Two previously published Illumina sequencing datasets were also aligned to the *C. jejuni* NCTC11168 genome for subsequent analysis and comparison with our dataset [16,21].

### RNA-seq fold change analysis

SAMMate with the edgeR statistical package was used to determine gene expression fold changes using the current Genbank protein annotation information for *C. jejuni* NCTC11168 [22,23].. In addition, we used SAMMate to calculate the RPKM values for all the known *C. jejuni* NCTC11168 ORFs. RPKM values normalize the number of reads aligning to a particular gene by taking into account the gene length and total number of reads that align to the genome. RPKM values can thus be compared across sequencing reactions and between genes to determine the relative expression of genes. Predicted pseudogenes and non-protein coding RNA elements were excluded from the fold change analysis to avoid introducing artifacts. Transcripts showing a fold change >= 1.5 with a *p* value <= 0.01 were considered to be significantly differentially expressed. 

### Absolute Transcript Profiling

The absolute transcript level of each gene in the *C. jejuni* genome was estimated using the calculated RPKM values (Table S4). Moreover the RPKM values of each gene were summed by on their Clusters of Orthologous Groups (COG) annotations. Genes that contain multiple COG annotations were included in each category and genes with no COG annotation were included in the “S – Function Unknown” category (Table S5). The overall transcriptomic contribution of each COG category under iron replete or iron limited conditions was compared using a two-way ANOVA with a Bonferroni post-test. Overall transcriptional differences of ≥1.5 fold with a *p* value < 0.05 were considered significant.

### *C. jejuni* NCTC11168 variant analysis

Aligned reads were used to determine if our NCTC11168 strain (hereafter denoted as WT^AS^) differed from the reference sequence [24,25]. Potential variant sites were called from the aligned reads using the mpileup and bcftools functions in Samtools [26]. It should be noted that Bowtie v0.12.7 does not support gapped alignment and thus potential indels were not identified using this approach. Sites varying from the reference sequence were then manually screened taking into account read depth and read qualities at each site. Consensus mutations were called if present in 4/5 sequencing runs with at least 10 aligned reads at the mutant location in each run and a mpileup consensus quality score >100. This analysis was also repeated using previously published *C. jejuni* NCTC11168 RNA-seq results [16]. The Chaudhuri et al. datasets consisted of a *C. jejuni* NCTC11168 strain and its isogenic *rpoN* deletion mutant and are denoted as WT^AG^ and Δ*rpoN* ^*AG*^ [16]. These results were compared with resequencing studies to identify common substitution mutations in NCTC11168 strains used in various different laboratories (Table 1) [21,27]. Cooper et al have recently re-sequenced the original NCTC11168 strain (NCTC11168-O or WT^Orig^) and compared this strain to the genome sequenced NCTC11168 (NCTC11168-GS or WT^Seq^) [27]. Jerome et al. have also resequenced a NCTC11168 isolate prior to mouse adaptation [21]. Only the pre-mouse adapted strain was considered (WT^LMp^) to avoid conflating mouse specific mutants. We also performed this analysis on the *C. jejuni* NCTC dRNA-seq datasets from the Dugar et al. study [17], however no consensus mutations from this dataset passed our cut-off values. 

**Table 1:** Common substitution mutations present in *C. jejuni* NCTC 11168 isolates from different laboratories.

**Location^1^**	**WT^Seq^**	**Mutation**	**Strain(s)**	**Gene**	**Consequence**
253191	A	G	All*	*mreB*	Ile to Val
253436	G	A	WT^AG^	*mreB*	Ala to Thr
262345	A	G	All*	*cheA*	Ile to Thr
263831	G	A	WT^AS^	*cheV*	Arg to Ser
393542	T	A	WT^Orig^, WT^LMp^	*Cj0431*	Stop to Lys
420550	A	G	WT^Orig^, WT^LMp^	*Cj0455c*	Stop to Glu
760188	A	G	All*	*Cj0807*	Lys to Glu
1189659	A	G	WT^LMp^	*porA*	Glu to Gly
1536694	A	G	WT^AG^	*cj1608*	Glu to Gly

1 All locations are based on the WT^Seq^ sequence

* WT^AS^, WT^Orig^, WT^LMp^, WT^AG^ and Δ*rpoN* ^AG^

### 5’UTR Identification

Aligned reads for all sequencing runs were visualized using Artemis [20]. Potential 5’UTRs were identified by manually inspecting reads that extended beyond the *C. jejuni* NCTC11168 predicted start codon [24,25]. 5’UTR coordinates and lengths were empirically assigned based on the presence of >3 reads with no potential overlap from reads extending from other ORFs or potential ncRNAs. Care was taken not to over infer the length/presence of 5'UTRs given the relative paucity of *C. jejuni* intergenic regions, along with the presence of many operonic transcripts. A similar 5’UTR screening was also done on the Chaudhuri et al. dataset [16]. This manual annotation was merged with the automated 5’UTR identification reported by Dugar et al. [17].

### *C. jejuni* genes in operonic structure

The average insert size of the various paired-end sequencing reaction was found to be ~200 bp (Figure S4). Aligned paired-end reads were used to infer the operonic structure of *C. jejuni* transcripts. Notably, this analysis relies on the active transcription of these operons at high enough levels to be detected accurately. As such, this analysis will undoubtedly underestimate the true number of co-transcribed genes and operonic transcripts present in *C. jejuni* and does not rule out the presence of alternative transcriptional start sites.The aligned reads were parsed using Bedtools [28] to identify reads that overlapped two sequential transcripts coded on the same strand by at least 50 bp into each gene. Genes were selected as potentially being in an operonic structure if they contained >20 reads aligning on both genes in at least two different sequencing samples under the same experimental conditions (iron replete/iron limited) (Table S8). Genes that were identified as co-transcribed were also compared with previously predicted operons using a bioinformatic approach [29]. Sequential genes that are present in operonic structure were merged together to form potential operonic transcripts (Table S9).

### Novel transcript Identification

Potential novel transcripts and antisense sRNAs were identified from the miRNA sequencing libraries. This approach would identify transcripts that would not be seen under normal Illumina library conditions due to the need for ~200 bp library fragment sizes. In addition, the use of strand specific sequencing in the miRNA library approach allowed for the identification of the actual transcript produced (as opposed to its complement). Both the positive and negative strand of the *C. jejuni* genome were screened for regions that contained >=10 reads aligning to either strand and that were at least 10 bp in length using BEDtools [28]. These regions were further refined by selecting sites that displayed a strand bias in their aligned reads (either >2 fold enrichment or no reads aligning to opposite strand). This analysis was done separately for both the iron replete and iron limited miRNA libraries and potential novel transcripts identified in each condition were subsequently merged together. Potential novel transcripts that were separated by <5 bp were considered to be contiguous transcripts and concatenated together. Regions were classified as potential antisense sRNAs if they were present within and transcribed antisense to known ORFs. Regions were classified as potential ncRNAs if they were present within intergenic regions and not within 50 bp of an ORF transcribed on the same strand. Several putative ncRNAs were identified adjacent (~300bp) to *C. jejuni* rRNA sites and were thus annotated as fragments from rRNA processing. In addition, reads emanating from the *C. jejuni* CRISPR region were annotated as CRISPR processed elements. The intergenic regions found to contain potential novel transcripts were also examined in the Chaudhuri et al. dataset to confirm their transcriptional presence and also compared to the list of potential novel transcripts previously bioinformatically identified in this study [16]. It is important to note that the transcript sizes identified in this analysis may be underestimates of true transcript sizes since the miRNA library construction has a bias towards smaller RNA fragments and our bioinformatic approach is stringent with regards to the number of reads required to identify sRNAs.

### Novel transcript RT-PCR and qRT-PCR

Validation of several putative novel transcripts was done using RT-PCR. Briefly, 5 µg of total RNA purified from iron limited conditions was subjected to reverse transcription using Superscript II (Invitrogen) using the manufacturers recommended protocol with the optional RNaseH treatment. A control reaction without the addition of Superscript II was also performed. PCR was then done using 2 µl of the first strand cDNA mixture and primers specific to each potential transcript. Primer sets known to amplify mRNA transcripts or genomic regions were also included to ensure the absence of contaminating genomic DNA.

The selected ncRNA regions were also tested by qRT-PCR to determine if they were differentially expressed under iron limitation as described previously [30]. In addition, these ncRNA transcripts were also tested to see if they were differentially expressed in a Δ*fur* mutant as compared to wildtype under iron replete conditions. Briefly, total RNA was purified from *C. jejuni* cultures grown under iron limited or iron replete conditions as described above. qRT-PCR was performed on an Applied Biosystems 7300 thermocycler using the QuantiTect SYBR Green RT-PCR Kit from Qiagen with the same primers as in the RT-PCR. Fold changes were determined according to the ΔΔC_T_ method using *slyD* as an endogenous control [31]. qRT-PCR was done on three independent RNA extractions with technical triplicates for each ncRNA tested. ncRNAs were considered differentially expressed if their expression was altered by >1.5-fold with *p*< 0.05. 

### Northern Blotting

Northern blotting was also used to validate the presence of one of our unique ncRNAs (ncRNA) using the DIG-Northern Starter kit from Roche and following the manufacturers instructions. Briefly, 1 µg of total RNA purified from *C. jejuni* NCTC 11168 grown under iron replete/limited conditions and the Δ*fur* mutant grown under iron replete conditions was loaded onto a 10% TBE-urea gel and ran for at 100V for 5 hours at 4°C. The RNA was then transferred to a nylon membrane overnight at 30mA at 4°C. DIG labelled probes for ncRNA_Z and *C. jejuni* 5S RNA were transcribed following the manufactuer’s instructions and using the indicated primer sets (5S_F acagatgtggaaacgccttg 5S_R TAATACGACTCACTATAGGGttaacaagtccgcaatgagc ncRNA_Z_F TAATACGACTCACTATAGGGGAGGGGTTTTTACCCCAAAG ncRNA_Z_R TGGGCAAGTAGGACAAAAGG). Probe hybridization was done using the manufacturer’s recommended temperatures and incubation times. 

## Results

### Identification of differentially expressed genes under conditions of iron limitation

**Figure 1:**
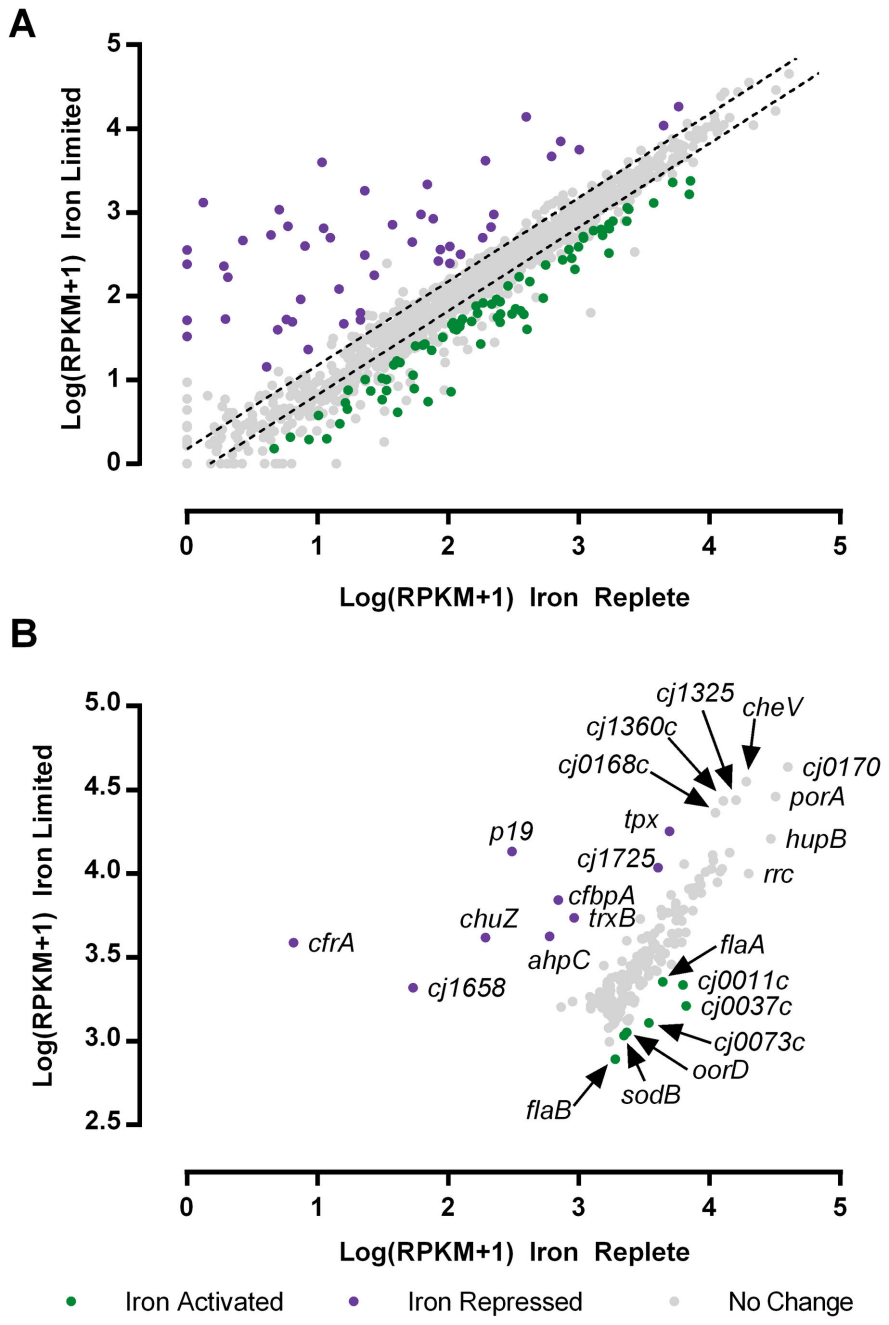
Differentially expressed genes under iron limitation. The average Log(RPKM+1) value for each gene was calculated under iron limited and iron replete conditions and plotted above. Significantly differentially expressed genes (p<0.01) are highlighted in green (iron activated) or purple (iron repressed) (Panel A). The hashed bars represent 1.5 fold differences in gene expression under each condition. The 90th percentile of the most highly expressed genes were plotted separately to compare the most highly expressed genes under iron limited and iron replete conditions (Panel B).

**Figure 2:**
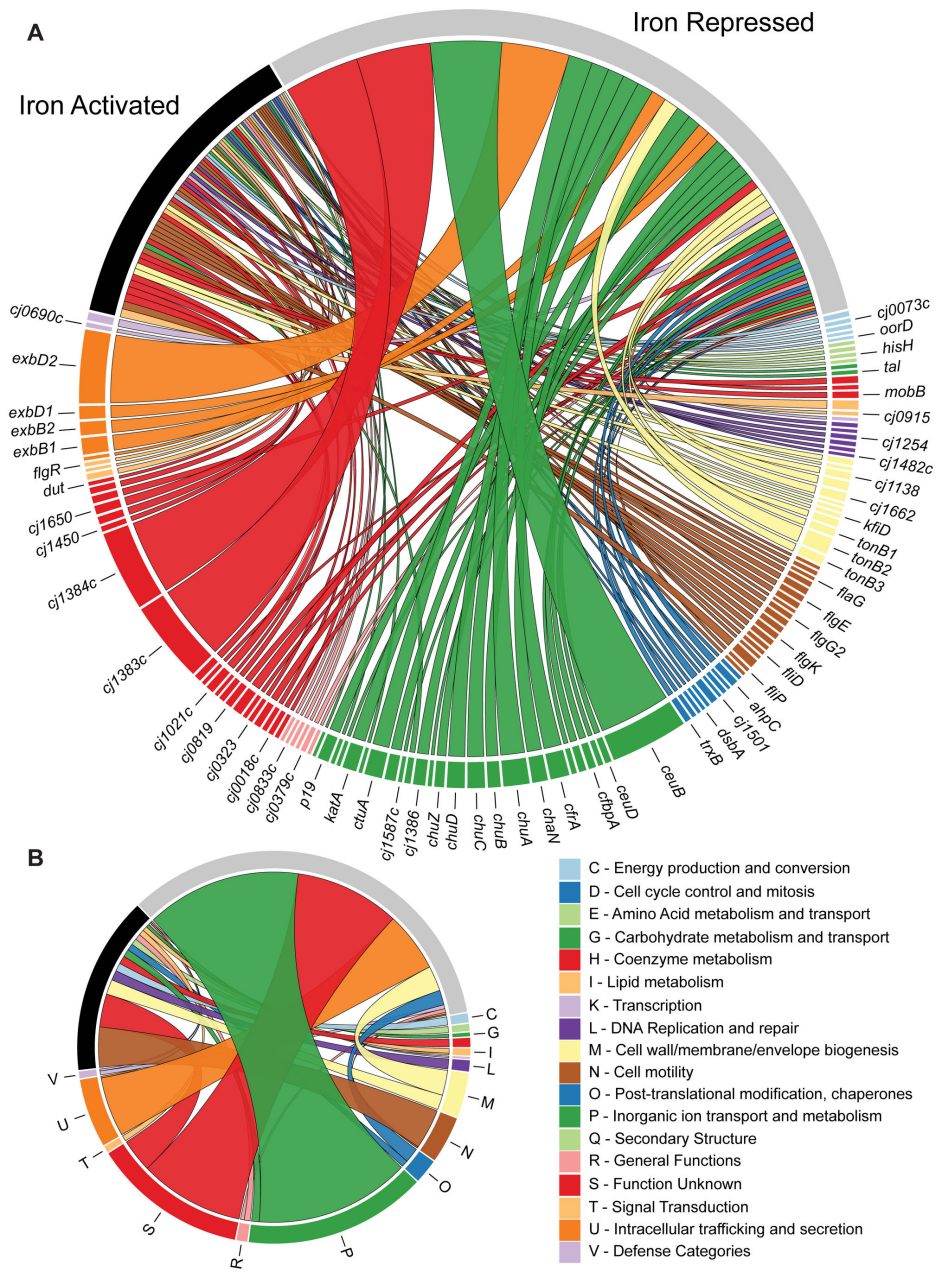
Iron activated and repressed genes by functional category. Individual genes that were found to be differentially expressed between iron limiting and iron replete conditions were sorted by COG category and plotted based on their degree of differential expression (Panel A). Genes that were differentially expressed were grouped by COG category and the overall COG category was plotted base on its degree of differential expression (Panel B). The size of the ribbon reflects the degree of activation/repression seen for each gene/COG category. Figure made using Circos v0.54 [52].

In order to identify iron regulated genes, the number of reads aligning to each protein coding gene and the corresponding RPKM value was determined using SAMMate [22]. The different sequencing runs were found to be highly reproducible, with R^2^ values ranging from 0.88-0.95 (Figure S1). The presence of significantly differentially expressed genes was determined using the edgeR statistical package [23]. This analysis determined that 127 genes were differentially expressed under iron replete/limited conditions with 77 iron activated genes and 50 iron repressed genes (Figure 1A, Figure 2 and Tables S2, S3). 

Iron repressed genes include almost all the known iron acquisition pathways in *C. jejuni* including the enterobactin (*cfrA, ceuBDE*) [4], hemin (*chuABCD, chuZ*) [32], lactoferrin/transferrin (*chaN/ctuA, cfbpABC*) [33] and rhodotorulic acid (*cj1658/p19,cj1660-1663*) transporters [2]. Genes with well-known roles in defense against oxidative stress were also iron repressed and include *katA, cj1386, ahpC, trxB* and *tpx*. In addition, most of the members of *C. jejuni*’s three TonB energy transduction systems (*exbB1/B2, exbD1/2, tonB1/B2/B3*) were iron repressed. Both the *cj1383c* and *cj1384c* genes located upstream from *katA* were highly differentially expressed under iron limitation. 

Many of the iron activated genes are involved in flagellar biogenesis. These include almost all the components of the filament (*flaA, flaB, flaG*, and *fliD*), hook (*flgE* and *flgE2*) and rod (*flgG, flgG2, flgH, flgI*, and *fliE*) flagellar structures. In addition, several flagellar chaperones (*flgD* and *fliK*) and regulatory proteins (*fliS, flgM*, and *flgR*) are also iron activated (Table S2). Finally, the transcript levels of the regulatory proteins Fur and CmeR are also increased under iron replete conditions, along with the expression of numerous genes involved in energy metabolism (such as *ccoQ, nuoM* and *cj0073c*).

The results from this analysis were compared to those previously obtained using microarray approaches under similar conditions [4]. While most of the RNA-seq iron repressed genes were identified under both experimental methods (37/50), a much smaller number of the RNA-seq identified iron activated genes were also found to be significantly differentially expressed by microarray (24/77) (Figure S2, Table S3). These novel iron activated genes include *fliS, flgB, flgD*, and *flgG*. Some genes were found to be iron activated in the RNA-seq but iron repressed in the microarray such as *sodB, modA, mobB* and *dsbA* (Table S3). In addition, most of the iron responsive genes have not been identified as being transcriptionally regulated (either directly or indirectly) by the Fur iron regulatory protein in *C. jejuni* (Figure S3) [4,10,30].

### Profiling absolute transcript levels under iron limitation

**Figure 3:**
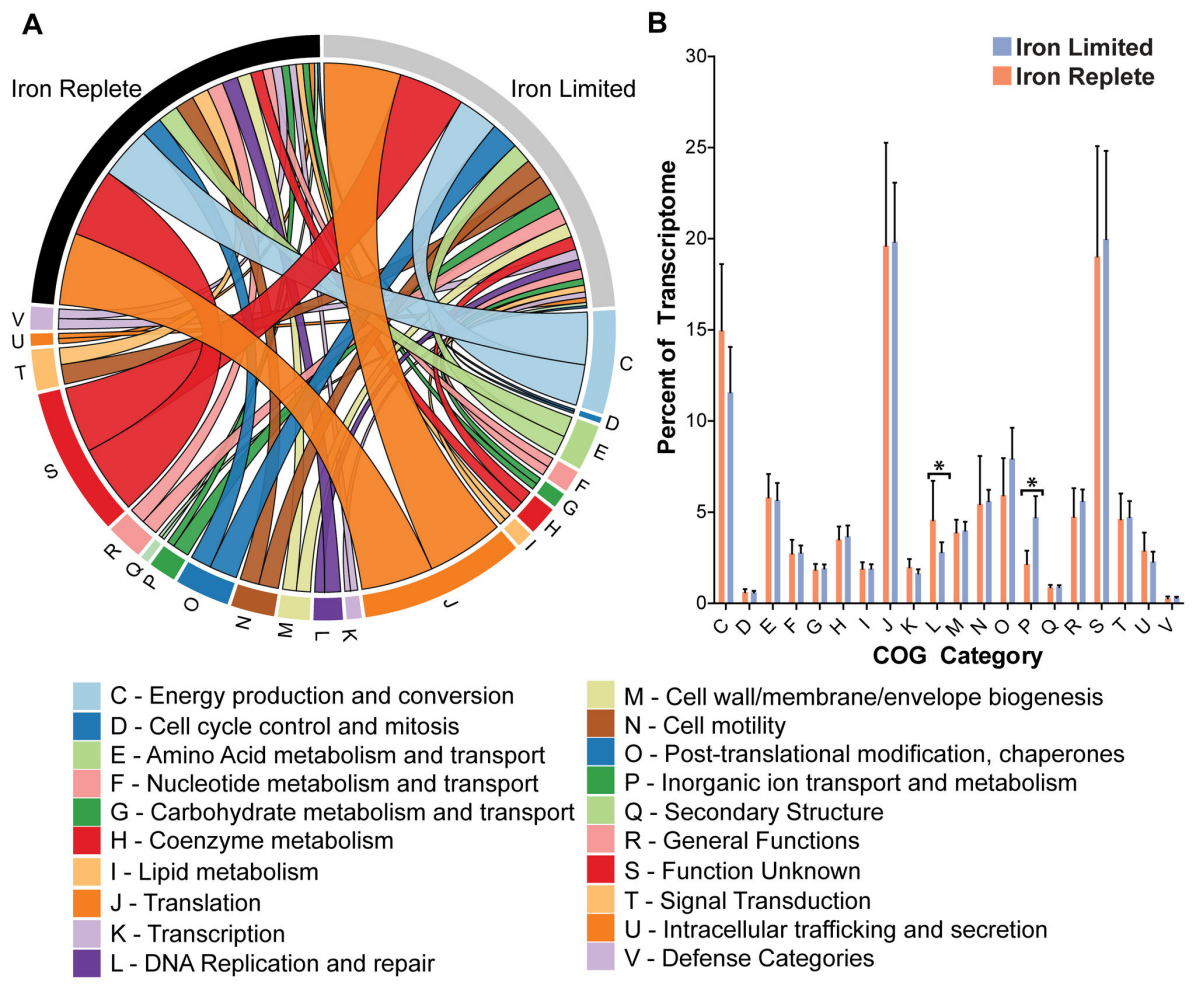
The overall transcriptome of *C. jejuni* is relatively unchanged under iron limitation. The absolute abundance of each gene in the *C*. *jejuni* genome was calculated and used to determine its contribution to the total transcriptome. These contributions were summed and grouped by COG category to determine the overall contribution of each COG category to the *C*. *jejuni* transcriptome under each condition (Panel A). The only statistically significant differences are enrichment for the COG category for inorganic ion transport and metabolism under iron limited conditions and enrichment for DNA replication and repair under iron replete conditions (Panel B) (* *p*<0.05). Figure made using Circos v0.54.

To estimate the absolute transcript abundance, the RPKM value for each gene was calculated and used to determine the percent contribution of each gene to the total transcriptional profile of the cell (Table S4) and to compute the 90^th^ percentile of expressed genes in either condition (Figure 1B). The most abundant transcripts in either iron state are very similar with *Cj0170*, *porA, cheV, cj1325, cj1360c, cj0168c and hupB* representing the most highly expressed genes in either condition (Figure 1B). As expected, the iron limited state is also characterized by relatively high expression of many iron acquisition genes including *p19, cfbpA, chuZ* and *cfrA*, while the iron replete state is characterized by the higher expression of flagellar (*flaA, flaB*) and energy metabolism (*cj0073c, oorD*) genes. The contribution of each COG category to the overall transcriptome was determined by grouping genes by COG function and summing their relative contributions (Table S5). As shown Figure 3 the overall COG transcriptional profile is quite similar between iron replete and limited conditions, with the exception of the COG categories for inorganic ion transport and metabolism, and DNA replication and repair, which were enriched in iron limited and iron replete conditions respectively (*p*<0.05).

### Analysis of *C. jejuni* NCTC11168 point mutations

Previous studies have highlighted the clonal complexity of *C. jejuni* and shown the contribution of point mutations to the physiology of *C. jejuni* NCTC11168. In order to define the isolate used in this study, we analyzed the RNA-seq data for the presence of point mutations present in our NCTC11168 strain as compared to the published genome-sequenced isolate. Importantly, the use of RNA-seq data to identify mutations is limited to the identification of nucleotide substitutions within protein coding regions in genes expressed at high enough levels to confidently call variants. Moreover, mutations that affect transcript abundance (transcription level or stability) may not be identified using this approach. Consensus mutations were also called in a conservative manner (see Methods) given the inherent increased error rate expected with this approach considering that the original genomic sequence was transcribed to mRNA, reverse transcribed to cDNA and subsequently PCR amplified. Nonetheless we were able to identify several consensus mutations present in our strain (WT^AS^) that differed from the reference NCTC11168 genome. These mutations were located in *mreB, cheA, cheV* and *cj0807* and all resulted in substitution mutations in their respective proteins. We also carried out a similar analysis of a previously published RNA-seq dataset (WT^AG^ and Δ*rpoN* ^*AG*^) [16]. These mutations were also compared with consensus mutations formerly found in NCTC11168 strains subjected to genome resequencing (WT^Orig^, WT^LMp^) [21,27]. Interestingly, analysis of WT^Orig^, WT^LMp^, WT^AG^ and Δ*rpoN* ^*AG*^ strains identified many of the same mutations in *mreB, cheA*, and *cj0807* that are present in the WT^AS^ strain, although the WT^AS^ strain appears to contain a unique mutation at base 26831 in *cheV* (Table 1). Several additional mutations originally identified in the WT^Ori^ and WT^LMp^ strains also appear to be present in the WT^AS^ strain but did not pass the stringent cutoffs we employed to avoid calling false positives. Our analysis also identified two additional unique mutations at bases 253436 and 1536694 in the WT^AG^ and Δ*rpoN* ^*AG*^ strains as compared to the other NCTC11168 strains (Table 1). 

### Genome wide identification of 5’UTRs

Given the potential role for 5’UTR in gene regulation, the presence and length of *C. jejuni* 5'UTRs were determined by inspecting the reads that aligned to the genome using the Artemis interface. This analysis was performed by combining the datasets generated in this study with the previously published *C. jejuni* RNA-seq dataset [16]. The results were then compared to the primary transcriptional start sites (TSS) identified by a recent dRNA-seq study [17]. Approximately ~400 putative 5'UTRs were identified from the two RNA-seq datasets (Table S6) as compared to the ~625 identified by dRNA-seq. The median 5'UTR length was 31 bases for both datasets, despite individual differences in the computed 5’UTR lengths of specific genes. Nearly 6.9% of the TSS identified in this study could be considered leaderless transcripts with a 5’ UTR shorter than 10 nucleotides as compared to 3.5% identified in the Dugar et al study [17]. 

### Genome wide identification of *C. jejuni* genes in operonic structure

In addition to identify gene boundaries, we have drawn on the paired-end sequencing information to identify the *C. jejuni* global operon structure. In total, 377 genes were determined to be in operonic structure using this analysis constituting 230 operons (Table S9). Some of these transcripts have been previously described, such as the *mfrABC* (formally *sdhABC*) [34], *cj1658-p19* [4] and *cj0399-fur-lysS-glyA-cj0403* [35] operonic transcripts. However, comparison of the identified transcripts with a previously published bioinformatic approach revealed the presence of numerous unpredicted operonic structures such as the co-transcription of *cj1420c*, *cj1421c*, *cj1422c* and *hddC*, *sodB* to *cj0170* and *cj0864* to *dsbB* [29].

### Identification and validation of novel transcripts

**Figure 4:**
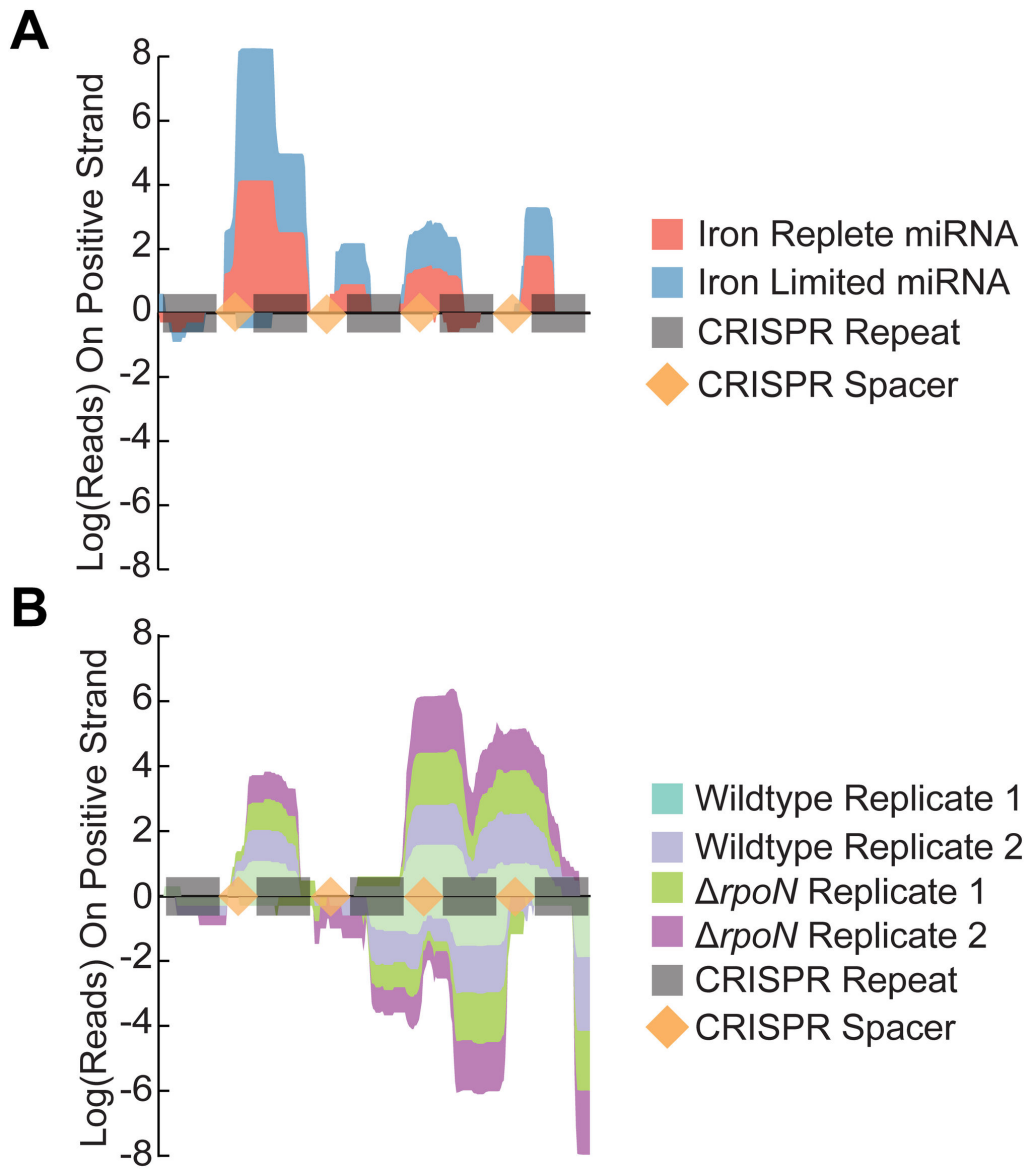
The CRISPR region is defined and strand specific in the miRNA sequencing library. Representation of the total number of reads mapping to the CRISPR region in either miRNA (Panel A) or standard (Panel B) Illumina sequencing libraries. Negative values refer to reads aligning on the negative strand. CRISPR repeat elements are highlighted in grey while the CRISPR spacers are in light orange.

The presence of novel transcripts and putative sRNAs was determined using the Illumina miRNA sequencing protocol to identify intergenic regions that displayed transcriptional activity. Analysis of the miRNA reads revealed the presence of widespread antisense RNA transcription throughout the *C. jejuni* genome with approximately 190 genes containing an antisense transcript (Table S7). The strand specificity and sensitivity of this approach was confirmed by analyzing the *C. jejuni* CRISPR region. Our analysis identified transcriptional activity from all five CRISPR elements present in the *C. jejuni* NCTC11168 genome in a strand specific manner (Figure 4). Moreover in most cases we could identify the individual processed CRISPR elements. 

**Figure 5:**
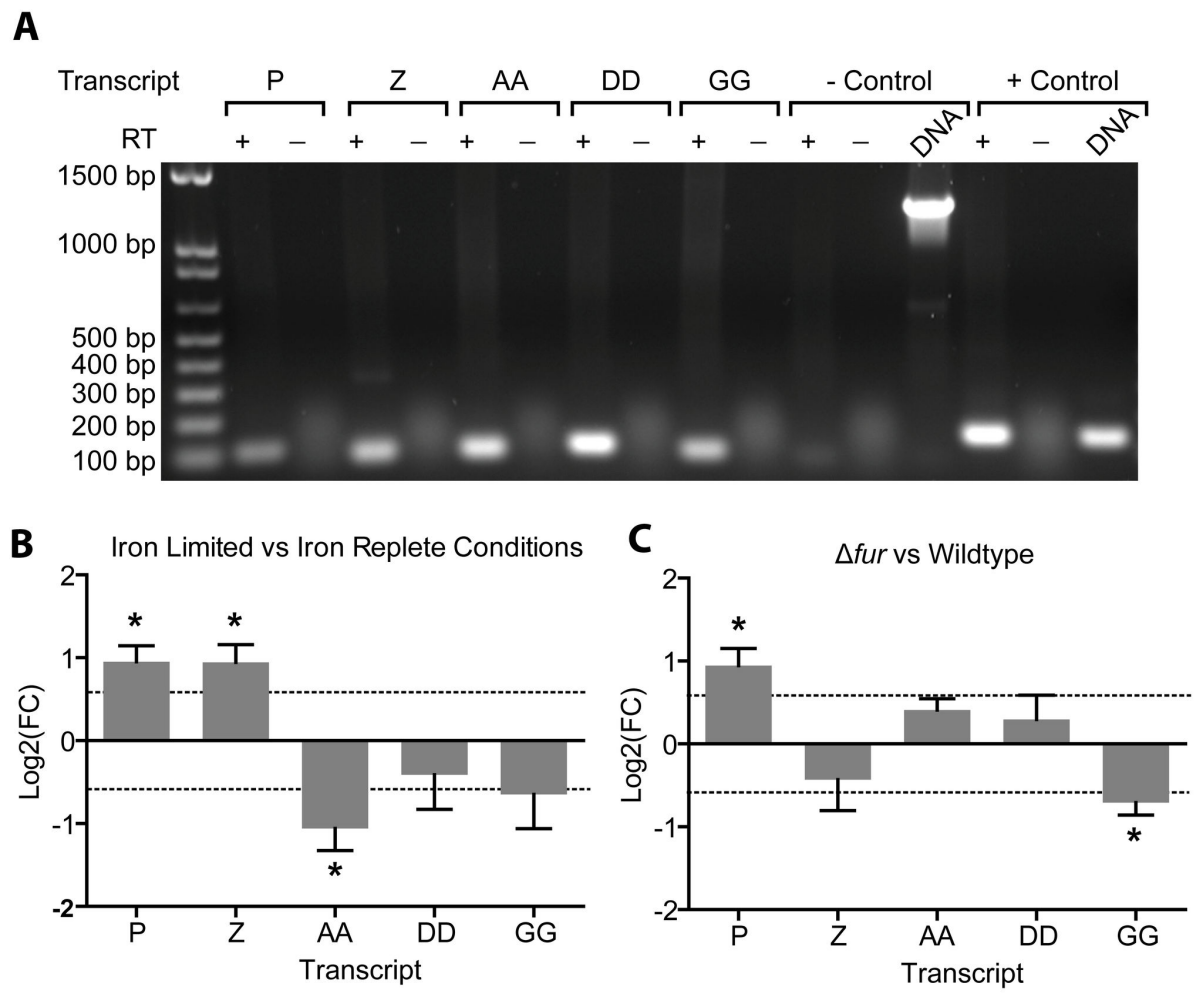
Regions containing identified novel transcripts are transcriptionally active and iron responsive in *C. jejuni.* RT-PCR was used to confirm the transcriptional activity of putative ncRNAs (Panel A). Templates marked + and - represent PCR reactions with and without reverse transcriptase respectively. RT-qPCR was used to determine if validated transcripts were responsive to iron (Panel B) or Fur regulation (Panel C) with a *p* value <0.05 (*).

Analysis of the miRNA reads also revealed the presence of 19 novel intergenic transcripts with several of these transcripts also detectable (albeit at very low levels) in the mRNA transcriptional analysis (Table S7). Selected novel transcripts were validated using RT-PCR confirming that these regions are transcriptionally active (Figure 5A). Several of these transcripts were also identified and validated using Northern blotting in a previous study [17]. Similarly, we used Northern blotting to confirm the expression of one of our unique ncRNAs (Figure S5A). In addition, the expression of these confirmed transcripts was quantified with qRT-PCR in both the wild-type and Δ*fur* mutant strains under iron replete and iron depleted conditions. The qRT-PCR results indicate that several of these novel transcripts are either Fur and/or iron responsive (Figure 5B). Of the five transcripts tested, three were found to be differentially expressed under iron limitation with ncRNA_P and ncRNA_Z showing iron repression and ncRNA_AA showing iron activation (Figure 5B). In addition ncRNA_P was found to be Fur repressed while ncRNA_GG was Fur activated (Figure 5C). The selected ncRNAs were found to be differentially expressed across varying concentrations of template RNA further validating our results (Figure S5B, S5C, S5D). 

## Discussion

Although iron homeostasis is now well established in *Campylobacter jejuni*, much remains to be understood about the regulatory mechanisms of the iron regulated genes. In this study, we combined RNA-seq and miRNA-seq approaches to catalogue all expressed transcripts from *Campylobacter jejuni* regardless of their structure or function under both iron replete and iron limited conditions. A total of 409 transcripts were characterized including 19 ncRNA. As compared to the previous transcriptomic studies in *C. jejuni*, our work has revealed 12 new ncRNAs and characterized the structure of 111 additional mRNA transcripts. These differences almost certainly arise from the differential expression of transcripts under different growth conditions emphasizing the regulation of sRNA expression in *C. jejuni*. Thus, our results further underscore the importance of cataloging bacterial transcripts under various conditions for a better understanding of the transcriptome of *C. jejuni* and its physiology. 

RNA sequencing of *C. jejuni* under iron limited conditions has underlined the importance of iron in modulating *C. jejuni*’s transcriptome. Iron limitation results in dramatic up regulation of almost all of *C. jejuni*’s iron acquisition systems including those for enterobactin, hemin and lactoferrin/transferrin [4,32,33]. Moreover, the vast majority of these genes are completely silenced under iron replete condition reflecting the tight regulation of these genes. This pattern of gene expression likely prevents iron overload and enables the expression of the right genes under the appropriate growth condition.

Interestingly, the most obvious feature of the genes that are iron activated is the preponderance of flagellar biogenesis genes. With the exception of the type III secretion system, almost all components of the *C. jejuni* flagella were found to be iron activated. This suggests that *C. jejuni* only up-regulates non-essential, but energetically costly processes such as flagellar biogenesis under periods of iron sufficiency. It is unclear exactly how *C. jejuni* couples flagellar gene expression with iron levels; however, previous studies have shown that several of these genes (*flaA, flaB, fliD*) appear to be directly regulated by the ferric uptake regulator Fur protein [30]. Moreover, several flagellar regulatory proteins (*fliS*, *flgR, flgM*) were also found to be iron activated. The activation of subsequent downstream effectors of *flgR* could thus be an indirect effect of high levels of iron in the cell. Given that motility is a well-known *C. jejuni* colonization factor, this suggests that there is an important regulatory coupling of flagellar biogenesis and iron levels in *C. jejuni*.

This study also provides new insight into *C. jejuni fur* regulation. Unlike other bacterial species, *C. jejuni fur* does not have its own promoter and also does not appear to autoregulate its own expression [35]. *C. jejuni fur* has been reported to be transcribed from two separate promoters that result in different polycistronic transcripts (with and without *gatC*) [35]. We were unable to detect the presence of a *gatC* containing *fur* polycistronic transcript by RNA-seq analysis and propose that the smaller transcript is the predominant form under our experimental conditions. Previous transcriptomic studies have reported that *C. jejuni fur* expression is not affected by iron levels [4,10], however in contrast to previous transcriptomic studies, our RNA-seq analysis revealed *fur* expression was found to be iron activated within *C. jejuni*. Intriguingly, while we identified *fur* to be iron responsive, none of the other members of its operon were found to be differentially expressed. This could be due to the fact that some of these genes are also transcribed as monocistronic transcripts in addition to their polycistronic forms [36]. 

While the set of iron-regulated genes we identified largely overlap with the set of genes previously identified by microarray analysis, the total number of differentially expressed genes is significantly different. Previous microarray results under similar growth conditions identified approximately 262 genes as being differentially expressed (>=2 fold change, *p* < 10^-4^) [4], while RNA-seq only identified 127 genes as being differentially expressed (Figure S2). Moreover, there was a marked bias in the number of genes that were identified as being either iron activated or iron repressed by either technique. Most of the iron repressed genes were identified using both techniques. However, many of the genes identified as being iron activated in the microarray were not differentially expressed in the RNA-seq. In addition, certain genes showed iron activation in the microarray while the RNA-seq found iron repression (Figure S2, Table S3). Some of these differences may be due to the slightly different growth conditions used in these two studies. These include the addition of 10 mM pyruvate to the media and the presence of 5% hydrogen in the atmospheric conditions for the RNA-seq study [4]. Previous reports have found that the addition of hydrogen changes the transcriptome of *C. jejuni* as compared to controls [37]. However, it is unclear why the addition of either pyruvate or hydrogen would preferentially alter gene expression in iron replete conditions as compared to iron limited conditions. One possibility is that pyruvate would act as a radical scavenger and reduce overall oxidative stress levels [38]. This could explain the differential expression seen for *sodB*, which was identified as being iron activated in the microarray analysis but iron repressed in the RNA-seq. Pyruvate and hydrogen could also alter the expression of metabolic genes [37]; indeed the most highly iron activated genes in the microarray are metabolic genes that were not found to be differentially expressed in the RNA-seq analysis. Another explanation for the discrepancy between the microarray results and the RNA-seq is that the additional replicates present in the microarray study provide more statistical power to identify differentially expressed genes. 

Notably, a recently published RNA-seq analysis in *C. jejuni* has found similar results with regard to the concordance between microarrays and RNA-seq [16]. This study re-analyzed the transcriptional profile of wild-type NCTC11168 as compared to its isogenic *rpoN* deletion mutant. This study also had to contend with transcriptional differences that could be due to slightly different growth conditions. Nonetheless, their analysis found that the previous microarray results [39] identified significantly more differentially expressed genes as compared to RNA-seq analysis. Intriguingly there was also a bias in the genes that were identified as being differentially expressed in the microarray but not in the RNA-seq. Most of the microarray identified *rpoN* activated genes were not identified in the RNA-seq data, while almost all of the *rpoN* repressed genes were present [16]. It remains to be seen whether this is a general phenomenon or specific to the conditions studied in these analyses. 

RNA-seq data can also be analyzed with regards to absolute transcript abundance as well as calculating fold changes. RPKM values were used to calculate the most abundant transcripts present under both iron replete and iron limiting conditions and also to determine the relative distribution of transcripts based on COG annotation (Figure 3, Tables S4, S5). The most abundant transcripts in either iron state are very similar with *Cj0170*, *porA, hupB, rrc* and *cheV* being amongst the most highly transcribed genes in the *Campylobacter* genome. It is somewhat humbling to note that there is almost nothing known about *Cj0170*, despite its place as the most highly expressed gene in the *C. jejuni* genome under both conditions. The high expression of *porA* is unsurprising given its abundance on *C. jejuni*’s surface. *HupB* encodes for DNA binding proteins and its high expression is probably related to this role [40]. *Rrc* encodes for an antioxidant protein and its high transcription probably points to its importance in oxidative stress defense [41]. Serine metabolism is critical for *C. jejuni* colonization [42] and *cheV*’s important role in chemotaxis towards serine is highlighted by its high expression level in both conditions [43]. The overall contribution of different COG categories to *C. jejuni*’s transcriptional profile remains relatively unchanged between iron replete and iron limited states (Figure 3). The sole exceptions to this are the category for inorganic ion transport and the category for DNA replication and repair. The overabundance of genes for inorganic ion transport in iron limited conditions is undoubtedly linked to the overexpression of iron acquisition genes. In particular, the periplasmic iron binding protein P19 becomes one of the most highly expressed transcripts under iron limitation. The overabundance of transcripts for DNA replication and repair could reflect the increased growth capacity of *C. jejuni* under iron replete conditions or decreased capacity under iron limited conditions. Interestingly, other categories that contain a large number of differentially expressed genes (i.e. motility genes) do not show an overall change in their transcriptome contribution. This outcome is primarily due to non-statistically significant transcript level changes in other members of the same category. In particular, some of the most highly transcribed genes are related to motility (such as *cheV*) and show non-statistically significant transcriptional decreases under iron rich conditions. These decreases in transcription by genes such as *cheV* cancel out the increases seen in many over expressed flagellar genes and result in no overall change in the COG category for motility. 

*C. jejuni* has long been known to rapidly mutate due to its lack of several DNA repair systems [44,45]. This raises the possibility that different labs working on the same strain could in fact be using variants that contain mutations in key genes. The presence of these variants could result in laboratories observing different phenotypes when working with the same strain. This has already been seen for the NCTC11168 strain with some laboratories reporting motility and the ability to colonize chicks and others reporting conflicting results [4,18,46,47]. We therefore conducted an analysis of potential point mutations present in both our laboratory NCTC11168 strain and the strain used for the *rpoN* RNA-seq profiling [16]. For simplicity we will refer to our NCTC11168 strain as WT^AS^ and the strains used in the *rpoN* RNA-seq study as WT^AG^ and Δ*rpoN* ^*AG*^. We also compared our results to NCTC11168 strains that had undergone resequencing studies (WT^Orig^, WT^LMp^). Our analysis found that all the RNA-seq sequenced strains (WT^AS^, WT^AG^, Δ*rpoN* ^*AG*^) contained the same point mutations as compared to the WT^Orig^ and WT^LMp^ strains (Table 1). Given the presence of these mutations in multiple independently sourced NCTC11168 strains, this suggests that these mutations are widespread within the *C. jejuni* research community. Further studies would be required to determine whether these mutations could result in functional differences between strains. This is especially true in the case of the *cheA* mutation given *cheA*’s known role in *C. jejuni* colonization [48]. Also, both the WT^AS^ and WT^AG^/Δ*rpoN* ^*AG*^ strains contain additional unique mutations as compared to the reference sequence. It should be noted that there may be additional mutations present within non-transcribed regions (i.e. promoter regions) in these strains which were not identifiable by RNA-seq. 

We also used our RNA-seq data along with the *rpoN* RNA-seq dataset to systematically identify 5’UTRs in *C. jejuni*. Potential 5’UTRs were manually annotated independently in each RNA-seq dataset and merged to provide a global map of *C. jejuni* 5’UTRs. When merging the datasets the longest 5’UTR identified was taken as the true 5’UTR. The merged dataset contains 420 identified 5’UTRs with a median length of 31 bases (Table S6). A recent study has documented *C. jejuni* NCTC 5’UTRs using dRNA-seq and as expected, identified additional and longer 5’UTRs due to dRNA-seq’s ability to enrich for unprocessed RNAs [17]. However, given that our dataset includes sequencing data from iron limited conditions, we were able to identify the 5’UTRs for many additional genes that are iron repressed and thus not expressed under the dRNA-seq study’s growth conditions. These include genes such as *cfrA, ctuA, chuA* and *tonB3*. 

The reads generated in our paired end RNA-seq study were relatively long in size with the average insert size for our libraries at approximately 200 bp in length (Figure S4). This allowed us to generate an operon map for the majority of *C. jejuni* genes. Adjacent genes that were identified as being in operonic structure were catalogued and compared to a previous genome wide bioinformatic approach for predicting operon structures [29]. Approximately 380 genes were identified as being co-transcribed. These operonic partners could be further stitched together to postulate the presence of 230 operonic transcripts. Overall there was high concordance between these two approaches with ~90% of the co-transcribed genes identified in this study correctly predicted using the bioinformatic approach. Finally, our data provides experimental evidence for multiple suspected operons including *sodB-cj0170, rrc-cj0011c* and *cj0864-dsbB*. Recent work using β-galactosidase assays has suggested that *cj0864-dsbB* share a common promoter but a direct test for operonic structure was not conducted [49]. 

We also used an alternate Illumina sequencing protocol to further depict the complete pool of novel transcripts present within *C. jejuni*. This approach allowed for the strand specific sequencing of small RNA fragments that would otherwise not be detected using conventional approaches. This can be demonstrated by comparing the read profiles over the CRISPR region in *C. jejuni*. CRISPR elements (crRNAs) are transcribed as one long unit that is then processed into the individual RNA fragments [50]. Standard Illumina library preparations can capture this preprocessed RNA fragment as seen in the Chaudhuri et al. results (our sequencing results showed minimal reads in this region) (Figure 4). In contrast, our miRNA sequencing protocol results in clear resolution of each of the individual processed CRISPR elements with minimal overlap. Our results indicate that the crRNAs each appear to be expressed at different levels. These differences could be due to certain crRNAs being more stable than others or point to the existence of alternate crRNA transcriptional start sites. Recent work by Dugar et al. has also found that the crRNAs are expressed at different levels and have proposed that each rRNA could be independently transcribed from their own promoter [17]. Our results agree with this model however the abundance of the individual crRNAs differs in our study as compared to Dugar et al... Under our experimental conditions the most abundant crRNA is crRNA2, while it is crRNA4 in the dRNA-seq study [17]. This difference suggests that *C. jejuni* may differentially regulate the expression of its crRNA’s based on environmental conditions.

Further analysis of the miRNA dataset revealed the presence of widespread anti-sense transcription in the *C. jejuni* genome. These antisense RNA species may prove to play important roles with regards to regulating gene expression in *C. jejuni*. In addition, our analysis identified numerous novel transcripts that were produced from intergenic regions. Several of these transcripts had been bioinformatically identified in the Chaudhuri et al. RNA-seq study [16] and were also identified by Dugar et al [17] using dRNA-seq (Table S7). Our experimental results also demonstrated that several of these ncRNAs are differentially expressed in response to iron and also appear to be regulated by the Fur transcriptional regulator (Figure 5B, 5C). These findings suggest that these putative ncRNAs may perform regulatory roles in iron homeostasis. Previous work by our lab has determined the CjFur regulon by both microarray and ChIP-chip analyses [4,30]. Comparing the RNA-seq iron responsive genes with the CjFur direct and indirect regulons reveals that most iron responsive genes in *C. jejuni* are not directly or indirectly regulated by CjFur (Figure S3)[2,4,30]. Specifically, the iron responsive ncRNA may be the regulatory link between genes that are iron responsive but not regulated by the Fur protein, while the Fur responsive ncRNAs may explain why numerous Fur regulated genes are indirectly, rather than directly, regulated by the Fur protein.

Searches in sRNA databases (such as Rfam) failed to identify homologous ncRNAs matching the novel sRNAs identified in this study. However, many bacterial ncRNAs are dependent on the Hfq RNA binding protein for full activity [3]. Since *C. jejuni* does not contain a homologue of Hfq, this could explain why *C. jejuni* sRNAs do not share structural/sequence homology to previously characterized sRNAs in other bacteria. A similar observation has been reported for *H. pylori*, with many identified sRNAs not matching known sRNA families [51]. It should also be noted that *C. jejuni* and *H. pylori* have exceptionally AT rich genomes that could also confound efforts to identify regions of similarity between sRNAs. It is also conceivable that additional novel RNAs remain to be identified in *C. jejuni* as our analysis did not detect some of the ncRNAs previously identified by dRNA-seq [17]. This failure to identify all potential ncRNAs may be due to our rigorous screening protocol (see methods). Indeed, our bioinformatic analysis excludes the *C. jejuni* 6S RNA transcript due to its proximity to known ORFs encoded on the same genomic strand [17,51].

In summary, our combined mRNA and miRNA Illumina library approaches have further defined our knowledge of *C. jejuni*’s transcriptomic response to iron limitation. Our RNA-seq study also provides a comprehensive operon map and a genome wide picture of the 5’UTR composition in *C. jejuni*. Finally, our miRNA sequencing approach has identified numerous uncharacterized novel RNAs within *C. jejuni* that are iron responsive and could thus play a role in iron homeostasis. Our results provide substantial novel insights into the transcriptional landscape of *C. jejuni* and thus constitute a valuable reference for *C. jejuni* researchers seeking to understand the transcriptional network of this model organism.

## Supporting Information

Figure S1 **Sequencing runs are highly similar.** The Log(RPKM+1) values obtained for each gene were compared across sequencing reactions from different biological replicates with the same growth condition. There is a high degree of similarity between different sequencing runs indicating low biological variability between samples.
(TIF)

Figure S2 **Plot of Log_2_(Fold Change) RNA-seq and microarray results.** The results of the RNA-seq in this study and previous microarray results [4] were plotted according to Log_2_(fold change). Genes identified as being iron repressed by both techniques are either dark purple (iron repressed) or dark green (iron activated). Genes only identified in the iron seq analysis are shown as light purple or green squares (repressed and activated respectively). Genes in blue were iron activated in the RNA-seq and iron repressed in the microarray. Genes in grey were not significantly differentially expressed in the RNA-seq analysis. See Table S2 for specific gene details.
(TIF)

Figure S3 **Comparison between Fur regulation and iron responsive genes.** The RNA-seq iron regulon was compared to the direct and indirect Fur regulons determined using ChIP-chip and microarrays respectively [4,53]. While the majority of the iron repressed genes were regulated by Fur, most iron activated genes were not Fur responsive. This suggests the presence of additional iron responsive regulatory elements in *C. jejuni*. The length of the bars/ribbons is proportional to the number of genes in each group. Figure made using Circos v0.54 [52].
(TIF)

Figure S4 **Average insert size of Illumina sequencing libraries.** The average insert size for each of the Illumina sequencing libraries constructed was calculated based on the paired-read alignment to the *C. jejuni* NCTC 11168 genome.
(TIF)

Figure S5 **Additional ncRNA validation experiments.** Panel A: Northern blot showing expression of ncRNA_Z across different conditions. The 5S RNA is also shown as a loading control. Densitometry analysis did not reveal differential expression of ncRNA_Z when normalized to the loading control. Panels BCD: qRT-PCR experiments of ncRNAs AA, Z and P with varying levels of template RNA for each RT reaction. Differentially expressed conditions (>1.5 fold change, *p* < 0.05) are indicated with an asterisk.
(TIF)

Table S1 **Statistics for high throughput sequencing runs.** Listed are the read statistics for the high throughput sequencing runs performed in this study as well as runs that were reanalyzed from other studies [16,21].
(XLSX)

Table S2 **Genes differentially expressed between iron limited and iron replete conditions.** Listed are the genes that were found to be significantly differentially expressed under iron limitation (>= 1.5 with a *p* value <= 0.01).
(XLSX)

Table S3 **Fold changes in gene expression as determined by RNA-seq and microarray analysis.** Listed are the fold changes in gene expression as determined by either RNA-seq or previous microarray analysis [4].
(XLSX)

Table S4 **RPKM values for each gene and sequencing run.** Listed are the RPKM values for each *C. jejuni* gene in each sequencing reaction.
(XLSX)

Table S5 **Percent contribution of COG categories to the total transcriptome of *C. jejuni*.** Listed are the overall contributions of each COG category to the total transcriptome.
(XLSX)

Table S6 **5'UTRs in *C. jejuni* as determined by RNA-seq.** Listed are the locations and lengths of the 5’UTR regions that were identified by manually inspecting reads that extended beyond the *C. jejuni* NCTC11168 predicted start codon. Included are those identified using previous a previous RNA-seq study [16] and those identified using dRNA-seq [17].
(XLSX)

Table S7 **Novel transcripts identified in *C. jejuni* as determined by RNA-seq.** Listed are the locations and strand origin of the novel *C. jejuni* NCTC11168 transcripts identified in this study.
(XLSX)

Table S8 **Genes Identified in Operonic Structure.** Listed are the genes identified as being in operonic structure by using the aligned RNA-seq paired-end reads to infer co-transcription.
(XLSX)

Table S9 **Operonic Transcripts in *C*.** ***jejuni***.
Listed are genes present in operonic structure that may form potential operonic transcripts.
(XLSX)

## References

[1] Andrews SC, Robinson AK, Rodríguez-Quiñones F (2003) Bacterial iron homeostasis. FEMS Microbiol Rev 27: 215-237. doi:10.1016/S0168-6445(03)00055-X. PubMed: 12829269. 12829269

[2] Butcher J, Flint A, Stahl M, Stintzi A (2010) *Campylobacter* Fur and PerR Regulons. In: Cornelis P Iron Uptake and Homeostasis in Microorgansims. Caister Academic Press.

[3] Massé E, Salvail H, Desnoyers G, Arguin M (2007) Small RNAs controlling iron metabolism. Curr Opin Microbiol 10: 140-145. doi:10.1016/j.mib.2007.03.013. PubMed: 17383226. 17383226

[4] Palyada K, Threadgill D, Stintzi A (2004) Iron acquisition and regulation in *Campylobacter* *jejuni* . J Bacteriol 186: 4714-4729. doi:10.1128/JB.186.14.4714-4729.2004. PubMed: 15231804. 15231804 PMC438614

[5] Miles S, Piazuelo MB, Semino-Mora C, Washington MK, Dubois A et al. (2010) Detailed *in* *vivo* analysis of the role of *Helicobacter* *pylori* Fur in colonization and disease. Infect Immun 78: 3073-3082. doi:10.1128/IAI.00190-10. PubMed: 20421381. 20421381 PMC2897407

[6] Simonsen KT, Nielsen G, Bjerrum JV, Kruse T, Kallipolitis BH et al. (2011) A role for the RNA chaperone Hfq in controlling adherent-invasive *Escherichia* *coli* colonization and virulence. PLOS ONE 6: e16387. doi:10.1371/journal.pone.0016387. PubMed: 21298102. 21298102 PMC3027648

[7] Fantappiè L, Metruccio MM, Seib KL, Oriente F, Cartocci E et al. (2009) The RNA chaperone Hfq is involved in stress response and virulence in *Neisseria* *meningitidis* and is a pleiotropic regulator of protein expression. Infect Immun 77: 1842-1853. doi:10.1128/IAI.01216-08. PubMed: 19223479. 19223479 PMC2681778

[8] Mey AR, Craig SA, Payne SM (2005) Characterization of *Vibrio* *cholerae* RyhB: the RyhB regulon and role of *ryhB* in biofilm formation. Infect Immun 73: 5706-5719. doi:10.1128/IAI.73.9.5706-5719.2005. PubMed: 16113288. 16113288 PMC1231101

[9] Oglesby AG, Murphy ER, Iyer VR, Payne SM (2005) Fur regulates acid resistance in *Shigella* *flexneri* via RyhB and *ydeP* . Mol Microbiol 58: 1354-1367. doi:10.1111/j.1365-2958.2005.04920.x. PubMed: 16313621. 16313621

[10] Holmes K, Mulholland F, Pearson BM, Pin C, McNicholl-Kennedy J et al. (2005) *Campylobacter* *jejuni* gene expression in response to iron limitation and the role of Fur. Microbiology 151: 243-257. doi:10.1099/mic.0.27412-0. PubMed: 15632442. 15632442

[11] van Vliet AH (2010) Next generation sequencing of microbial transcriptomes: challenges and opportunities. FEMS Microbiol Lett 302: 1-7. doi:10.1111/j.1574-6968.2009.01767.x. PubMed: 19735299. 19735299

[12] Mooney M, Bond J, Monks N, Eugster E, Cherba D et al. (2013) Comparative RNA-Seq and microarray analysis of gene expression changes in B-cell lymphomas of Canis familiaris. PLOS ONE 8: e61088. doi:10.1371/journal.pone.0061088. PubMed: 23593398. 23593398 PMC3617154

[13] Sîrbu A, Kerr G, Crane M, Ruskin HJ (2012) RNA-Seq vs dual- and single-channel microarray data: sensitivity analysis for differential expression and clustering. PLOS ONE 7: e50986. doi:10.1371/journal.pone.0050986. PubMed: 23251411. 23251411 PMC3518479

[14] Mutz KO, Heilkenbrinker A, Lönne M, Walter JG, Stahl F (2013) Transcriptome analysis using next-generation sequencing. Curr Opin Biotechnol 24: 22-30. doi:10.1016/j.copbio.2013.05.026. PubMed: 23020966. 23020966

[15] Wang Z, Gerstein M, Snyder M (2009) RNA-Seq: a revolutionary tool for transcriptomics. Nat Rev Genet 10: 57-63. doi:10.1038/nrg2484. PubMed: 19015660. 19015660 PMC2949280

[16] Chaudhuri RR, Yu L, Kanji A, Perkins TT, Gardner PP et al. (2011) Quantitative RNA-seq analysis of the Campylobacter jejuni transcriptome. Microbiology 157: 2922-2932. doi:10.1099/mic.0.050278-0. PubMed: 21816880. 21816880 PMC3353397

[17] Dugar G, Herbig A, Förstner KU, Heidrich N, Reinhardt R et al. (2013) High-resolution transcriptome maps reveal strain-specific regulatory features of multiple Campylobacter jejuni isolates. PLOS Genet 9: e1003495 PubMed: 23696746. 23696746 10.1371/journal.pgen.1003495 PMC3656092

[18] Gaynor EC, Cawthraw S, Manning G, MacKichan JK, Falkow S et al. (2004) The genome-sequenced variant of *Campylobacter* *jejuni* NCTC 11168 and the original clonal clinical isolate differ markedly in colonization, gene expression, and virulence-associated phenotypes. J Bacteriol 186: 503-517. doi:10.1128/JB.186.2.503-517.2004. PubMed: 14702320. 14702320 PMC305761

[19] Langmead B, Trapnell C, Pop M, Salzberg SL (2009) Ultrafast and memory-efficient alignment of short DNA sequences to the human genome. Genome Biol 10: R25. doi:10.1186/gb-2009-10-3-r25. PubMed: 19261174. 19261174 PMC2690996

[20] Carver T, Harris SR, Berriman M, Parkhill J, McQuillan JA (2012) Artemis: an integrated platform for visualization and analysis of high-throughput sequence-based experimental data. Bioinformatics 28: 464-469. doi:10.1093/bioinformatics/btr703. PubMed: 22199388. 22199388 PMC3278759

[21] Jerome JP, Bell JA, Plovanich-Jones AE, Barrick JE, Brown CT et al. (2011) Standing genetic variation in contingency loci drives the rapid adaptation of Campylobacter jejuni to a novel host. PLOS ONE 6: e16399. doi:10.1371/journal.pone.0016399. PubMed: 21283682. 21283682 PMC3025981

[22] Xu G, Deng N, Zhao Z, Judeh T, Flemington E et al. (2011) SAMMate: a GUI tool for processing short read alignments in SAM/BAM format. Source Code. Biol Med 6: 2. 10.1186/1751-0473-6-2 PMC3027120 21232146

[23] Robinson MD, McCarthy DJ, Smyth GK (2010) edgeR: a Bioconductor package for differential expression analysis of digital gene expression data. Bioinformatics 26: 139-140. doi:10.1093/bioinformatics/btp616. PubMed: 19910308. 19910308 PMC2796818

[24] Gundogdu O, Bentley SD, Holden MT, Parkhill J, Dorrell N et al. (2007) Re-annotation and re-analysis of the *Campylobacter* *jejuni* NCTC11168 genome sequence. BMC Genomics 8: 162. doi:10.1186/1471-2164-8-162. PubMed: 17565669. 17565669 PMC1899501

[25] Parkhill J, Wren BW, Mungall K, Ketley JM, Churcher C et al. (2000) The genome sequence of the food-borne pathogen *Campylobacter* *jejuni* reveals hypervariable sequences. Nature 403: 665-668. doi:10.1038/35001088. PubMed: 10688204. 10688204

[26] Li H, Handsaker B, Wysoker A, Fennell T, Ruan J et al. (2009) The Sequence Alignment/Map format and SAMtools. Bioinformatics 25: 2078-2079. doi:10.1093/bioinformatics/btp352. PubMed: 19505943. 19505943 PMC2723002

[27] Cooper KK, Cooper MA, Zuccolo A, Joens LA (2012) Re-sequencing of a virulent strain of *Campylobacter* *jejuni* NCTC11168 reveals potential virulence factors. Res Microbiol, 164: 6–11. PubMed: 23046762. 23046762 10.1016/j.resmic.2012.10.002

[28] Quinlan AR, Hall IM (2010) BEDTools: a flexible suite of utilities for comparing genomic features. Bioinformatics 26: 841-842. doi:10.1093/bioinformatics/btq033. PubMed: 20110278. 20110278 PMC2832824

[29] Price MN, Huang KH, Alm EJ, Arkin AP (2005) A novel method for accurate operon predictions in all sequenced prokaryotes. Nucleic Acids Res 33: 880-892. doi:10.1093/nar/gki232. PubMed: 15701760. 15701760 PMC549399

[30] Butcher J, Sarvan S, Brunzelle JS, Couture JF, Stintzi A (2012) Structure and regulon of *Campylobacter* *jejuni* ferric uptake regulator Fur define apo-Fur regulation. Proc Natl Acad Sci U S A 109: 10047-10052. doi:10.1073/pnas.1118321109. PubMed: 22665794. 22665794 PMC3382491

[31] Ritz M, Garenaux A, Berge M, Federighi M (2009) Determination of *rpoA* as the most suitable internal control to study stress response in *C.* *jejuni* by RT-qPCR and application to oxidative stress. J Microbiol Methods 76: 196-200. doi:10.1016/j.mimet.2008.10.014. PubMed: 19041906. 19041906

[32] Ridley KA, Rock JD, Li Y, Ketley JM (2006) Heme utilization in *Campylobacter* *jejuni* . J Bacteriol 188: 7862-7875. doi:10.1128/JB.00994-06. PubMed: 16980451. 16980451 PMC1636299

[33] Miller CE, Rock JD, Ridley KA, Williams PH, Ketley JM (2008) Utilization of lactoferrin-bound and transferrin-bound iron by *Campylobacter* *jejuni* . J Bacteriol 190: 1900-1911. doi:10.1128/JB.01761-07. PubMed: 18203832. 18203832 PMC2258864

[34] Guccione E, Hitchcock A, Hall SJ, Mulholland F, Shearer N et al. (2010) Reduction of fumarate, mesaconate and crotonate by Mfr, a novel oxygen-regulated periplasmic reductase in *Campylobacter* *jejuni* . Environ Microbiol 12: 576-591. doi:10.1111/j.1462-2920.2009.02096.x. PubMed: 19919540. 19919540

[35] van Vliet AH, Rock JD, Madeleine LN, Ketley JM (2000) The iron-responsive regulator Fur of *Campylobacter* *jejuni* is expressed from two separate promoters. FEMS Microbiol Lett 188: 115-118. doi:10.1111/j.1574-6968.2000.tb09180.x. PubMed: 10913692. 10913692

[36] Chan VL, Bingham HL (1991) Complete sequence of the *Campylobacter* *jejuni* *glyA* gene encoding serine hydroxymethyltransferase. Gene 101: 51-58. doi:10.1016/0378-1119(91)90223-X. PubMed: 2060796. 2060796

[37] John A, Connerton PL, Cummings N, Connerton IF (2011) Profound differences in the transcriptome of *Campylobacter* *jejuni* grown in two different, widely used, microaerobic atmospheres. Res Microbiol 162: 410-418. doi:10.1016/j.resmic.2011.02.004. PubMed: 21320592. 21320592

[38] Verhoeff-Bakkenes L, Arends AP, Snoep JL, Zwietering MH, de Jonge R (2008) Pyruvate relieves the necessity of high induction levels of catalase and enables *Campylobacter* *jejuni* to grow under fully aerobic conditions. Lett Appl Microbiol 46: 377-382. doi:10.1111/j.1472-765X.2008.02326.x. PubMed: 18266640. 18266640

[39] Kamal N, Dorrell N, Jagannathan A, Turner SM, Constantinidou C et al. (2007) Deletion of a previously uncharacterized flagellar-hook-length control gene *fliK* modulates the σ^54^-dependent regulon in *Campylobacter* *jejuni* . Microbiology 153: 3099-3111. doi:10.1099/mic.0.2007/007401-0. PubMed: 17768253. 17768253

[40] Konkel ME, Marconi RT, Mead DJ, Cieplak W Jr. (1994) Cloning and expression of the hup encoding a histone-like protein of *Campylobacter* *jejuni* . Gene 146: 83-86. doi:10.1016/0378-1119(94)90837-0. PubMed: 8063109. 8063109

[41] Yamasaki M, Igimi S, Katayama Y, Yamamoto S, Amano F (2004) Identification of an oxidative stress-sensitive protein from *Campylobacter* *jejuni*, homologous to rubredoxin oxidoreductase/rubrerythrin. FEMS Microbiol Lett 235: 57-63. doi:10.1111/j.1574-6968.2004.tb09567.x. PubMed: 15158262. 15158262

[42] Velayudhan J, Kelly DJ (2002) Analysis of gluconeogenic and anaplerotic enzymes in *Campylobacter* *jejuni*: an essential role for phosphoenolpyruvate carboxykinase. Microbiology 148: 685-694. PubMed: 11882702. 11882702 10.1099/00221287-148-3-685

[43] Hartley-Tassell LE, Shewell LK, Day CJ, Wilson JC, Sandhu R et al. (2010) Identification and characterization of the aspartate chemosensory receptor of *Campylobacter* *jejuni* . Mol Microbiol 75: 710-730. PubMed: 20025667. 20025667 10.1111/j.1365-2958.2009.07010.x

[44] Gaasbeek EJ, van der Wal FJ, van Putten JP, de Boer P, van der Graaf-van Bloois L et al. (2009) Functional characterization of excision repair and RecA-dependent recombinational DNA repair in *Campylobacter* *jejuni* . J Bacteriol 191: 3785-3793. doi:10.1128/JB.01817-08. PubMed: 19376866. 19376866 PMC2698403

[45] Han J, Sahin O, Barton YW, Zhang Q (2008) Key role of Mfd in the development of fluoroquinolone resistance in *Campylobacter* *jejuni* . PLOS Pathog 4: e1000083 PubMed: 18535657. 18535657 10.1371/journal.ppat.1000083 PMC2390758

[46] Jones MA, Marston KL, Woodall CA, Maskell DJ, Linton D et al. (2004) Adaptation of *Campylobacter* *jejuni* NCTC11168 to high-level colonization of the avian gastrointestinal tract. Infect Immun 72: 3769-3776. doi:10.1128/IAI.72.7.3769-3776.2004. PubMed: 15213117. 15213117 PMC427441

[47] Ahmed IH, Manning G, Wassenaar TM, Cawthraw S, Newell DG (2002) Identification of genetic differences between two *Campylobacter* *jejuni* strains with different colonization potentials. Microbiology 148: 1203-1212. PubMed: 11932464. 11932464 10.1099/00221287-148-4-1203

[48] Chang C, Miller JF (2006) *Campylobacter* *jejuni* colonization of mice with limited enteric flora. Infect Immun 74: 5261-5271. doi:10.1128/IAI.01094-05. PubMed: 16926420. 16926420 PMC1594848

[49] Grabowska AD, Wandel MP, Łasica AM, Nesteruk M, Roszczenko P et al. (2011) *Campylobacter* *jejuni* dsb gene expression is regulated by iron in a Fur-dependent manner and by a translational coupling mechanism. BMC Microbiol 11: 166. doi:10.1186/1471-2180-11-166. PubMed: 21787430. 21787430 PMC3167755

[50] Marraffini LA, Sontheimer EJ (2010) CRISPR interference: RNA-directed adaptive immunity in bacteria and archaea. Nat Rev Genet 11: 181-190. doi:10.1038/ni0310-181. PubMed: 20125085. 20125085 PMC2928866

[51] Sharma CM, Hoffmann S, Darfeuille F, Reignier J, Findeiss S et al. (2010) The primary transcriptome of the major human pathogen *Helicobacter* *pylori* . Nature 464: 250-255. doi:10.1038/nature08756. PubMed: 20164839. 20164839

[52] Krzywinski M, Schein J, Birol I, Connors J, Gascoyne R et al. (2009) Circos: an information aesthetic for comparative genomics. Genome Res 19: 1639-1645. doi:10.1101/gr.092759.109. PubMed: 19541911. 19541911 PMC2752132

[53] Butcher J, Sarvan S, Brunzelle JS, Couture JF, Stintzi A (2012) Structure and regulon of Campylobacter jejuni ferric uptake regulator Fur define apo-Fur regulation. Proc Natl Acad Sci U S A. PubMed: 22665794 10.1073/pnas.1118321109 PMC3382491 22665794

