# Supplementary figures and images for: The Transcriptional Landscape of Campylobacter jejuni under Iron Replete and Iron Limited Growth Conditions
Source: PLoS One. 2013 Nov 1;8(11):e79475. doi: 10.1371/journal.pone.0079475 (PMC3815106; doi:10.1371/journal.pone.0079475)

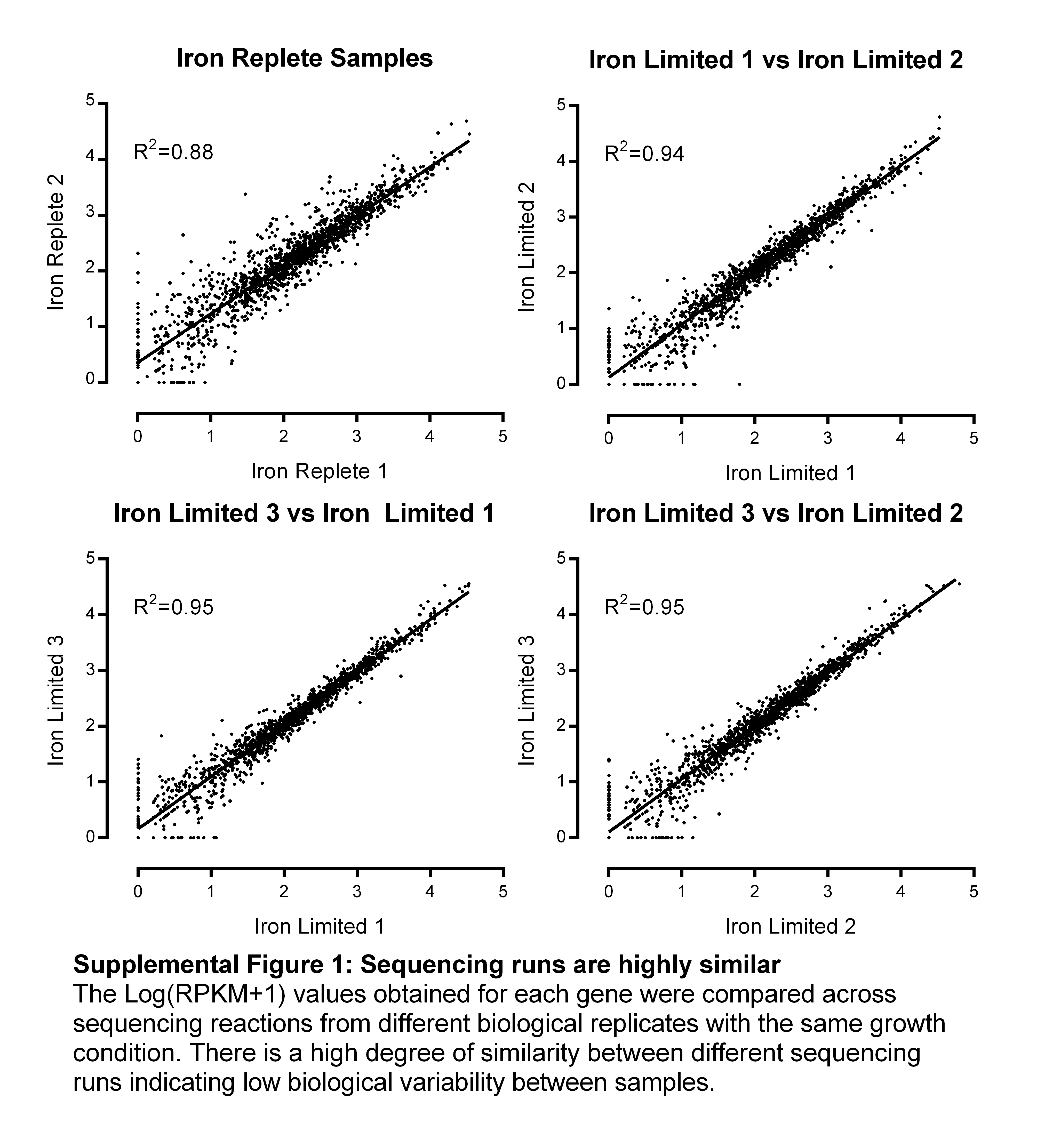

Supplement: Figure S1 — Sequencing runs are highly similar. The Log(RPKM+1) values obtained for each gene were compared across sequencing reactions from different biological replicates with the same growth condition. There is a high degree of similarity between different sequencing runs indicating low biological variability between samples. (TIF) [file pone.0079475.s001.tif]

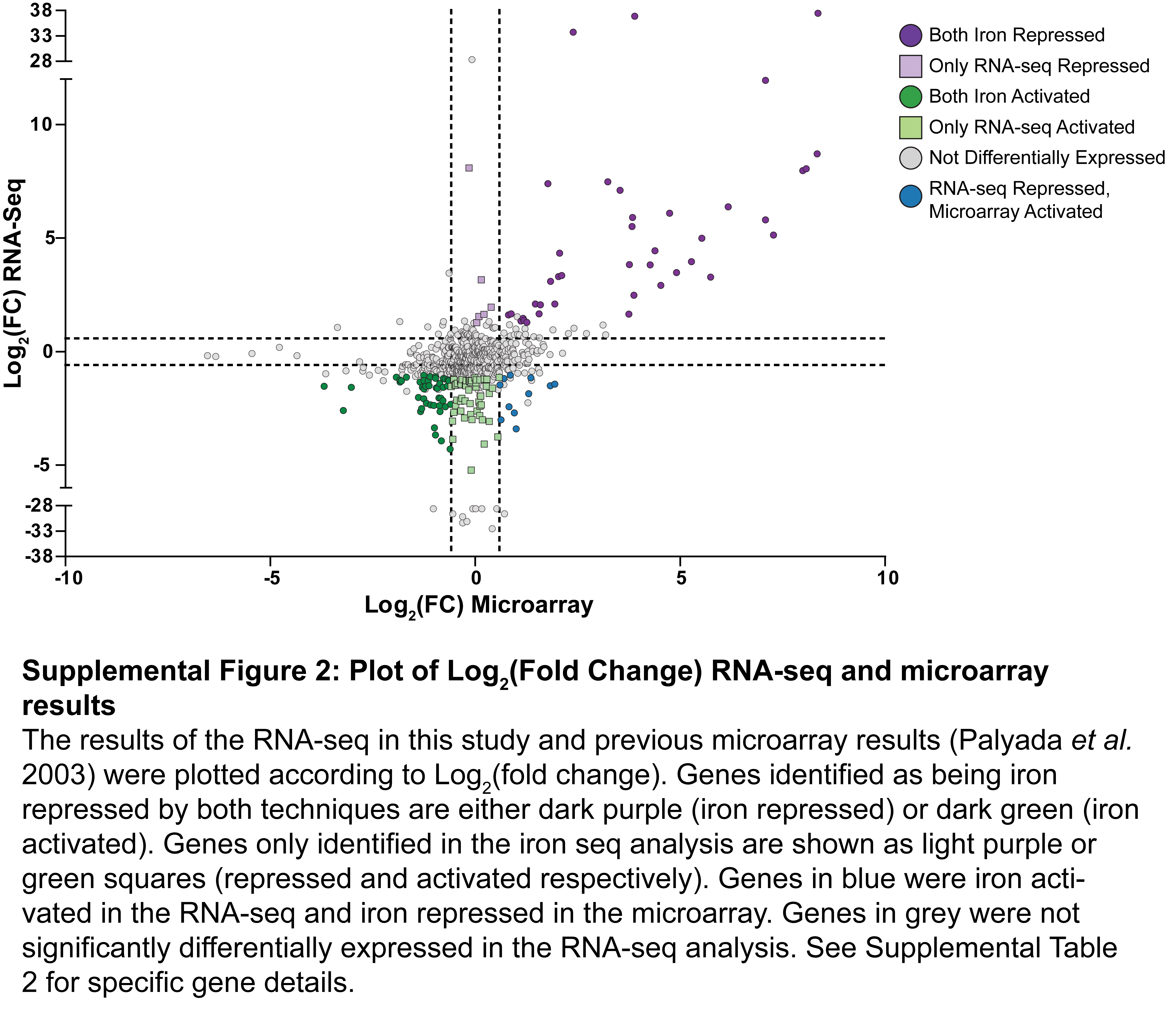

Supplement: Figure S2 — Plot of Log2(Fold Change) RNA-seq and microarray results. The results of the RNA-seq in this study and previous microarray results [4] were plotted according to Log2(fold change). Genes identified as being iron repressed by both techniques are either dark purple (iron repressed) or dark green (iron activated). Genes only identified in the iron seq analysis are shown as light purple or green squares (repressed and activated respectively). Genes in blue were iron activated in the RNA-seq and iron repressed in the microarray. Genes in grey were not significantly differentially expressed in the RNA-seq analysis. See Table S2 for specific gene details. (TIF) [file pone.0079475.s002.tif]

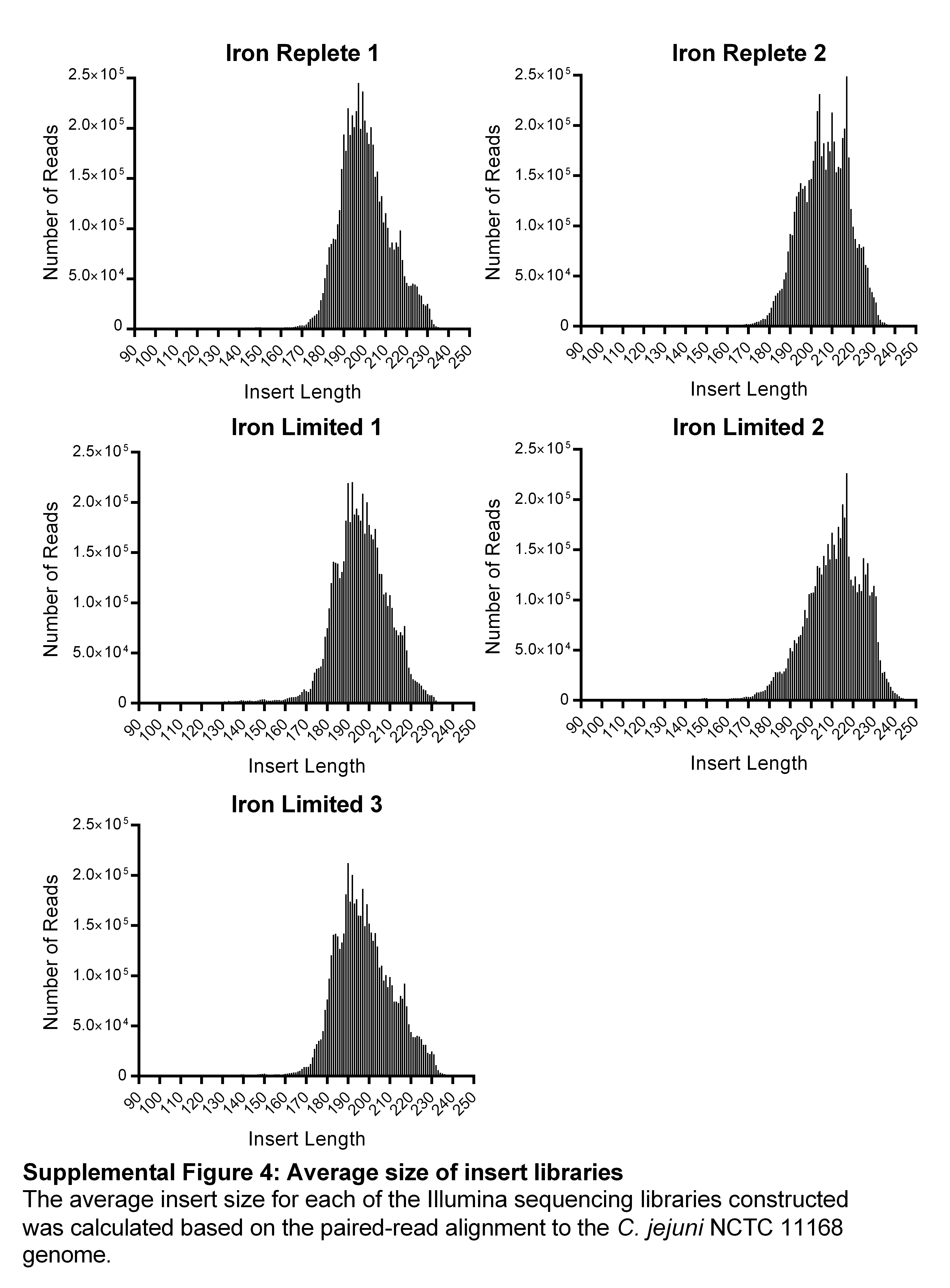

Supplement: Figure S4 — Average insert size of Illumina sequencing libraries. The average insert size for each of the Illumina sequencing libraries constructed was calculated based on the paired-read alignment to the C. jejuni NCTC 11168 genome. (TIF) [file pone.0079475.s004.tif]

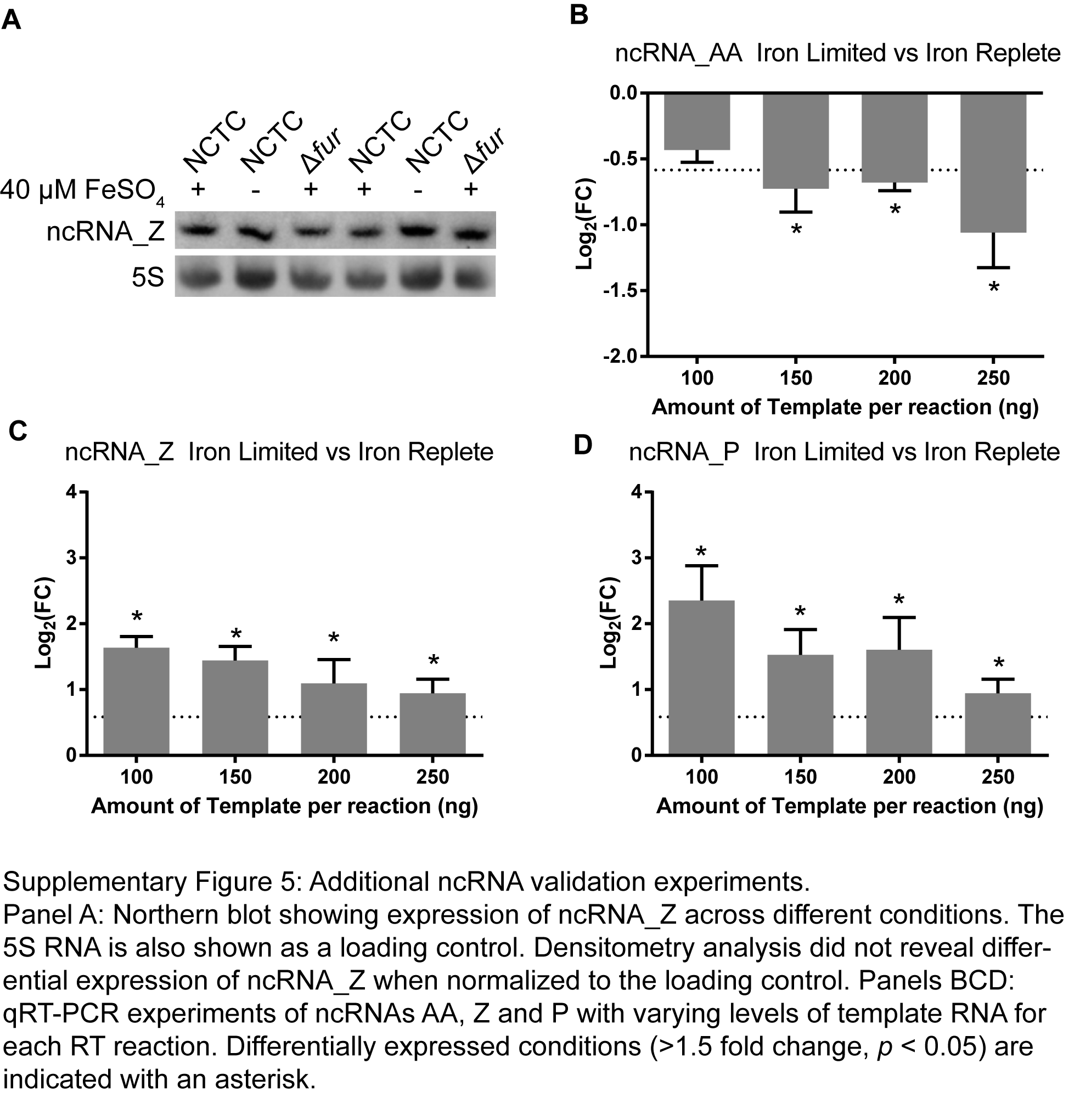

Supplement: Figure S5 — Additional ncRNA validation experiments. Panel A: Northern blot showing expression of ncRNA_Z across different conditions. The 5S RNA is also shown as a loading control. Densitometry analysis did not reveal differential expression of ncRNA_Z when normalized to the loading control. Panels BCD: qRT-PCR experiments of ncRNAs AA, Z and P with varying levels of template RNA for each RT reaction. Differentially expressed conditions (>1.5 fold change, p < 0.05) are indicated with an asterisk. (TIF) [file pone.0079475.s005.tif]
